# Complete genome sequence of *Arcobacter nitrofigilis* type strain (CI^T^)

**DOI:** 10.4056/sigs.912121

**Published:** 2010-06-15

**Authors:** Amrita Pati, Sabine Gronow, Alla Lapidus, Alex Copeland, Tijana Glavina Del Rio, Matt Nolan, Susan Lucas, Hope Tice, Jan-Fang Cheng, Cliff Han, Olga Chertkov, David Bruce, Roxanne Tapia, Lynne Goodwin, Sam Pitluck, Konstantinos Liolios, Natalia Ivanova, Konstantinos Mavromatis, Amy Chen, Krishna Palaniappan, Miriam Land, Loren Hauser, Yun-Juan Chang, Cynthia D. Jeffries, John C. Detter, Manfred Rohde, Markus Göker, James Bristow, Jonathan A. Eisen, Victor Markowitz, Philip Hugenholtz, Hans-Peter Klenk, Nikos C. Kyrpides

**Affiliations:** 1 DOE Joint Genome Institute, Walnut Creek, California, USA; 2 Los Alamos National Laboratory, Bioscience Division, Los Alamos, New Mexico, USA; 3 DSMZ – German Collection of Microorganisms and Cell Cultures GmbH, Braunschweig, Germany; 4 Biological Data Management and Technology Center, Lawrence Berkeley National Laboratory, Berkeley, California, USA; 5 Oak Ridge National Laboratory, Oak Ridge, Tennessee, USA; 6 HZI – Helmholtz Centre for Infection Research, Braunschweig, Germany; 7 University of California Davis Genome Center, Davis, California, USA

**Keywords:** symbiotic, *Spartina alterniflora* Loisel, nitrogen fixation, micro-anaerophilic, motile, *Campylobacteraceae*, GEBA

## Abstract

*Arcobacter nitrofigilis* (McClung *et al.* 1983) Vandamme *et al.* 1991 is the type species of the genus *Arcobacter* in the family *Campylobacteraceae* within the  *Epsilonproteobacteria*. The species was first described in 1983 as *Campylobacter nitrofigilis* [1] after its detection as a free-living, nitrogen-fixing *Campylobacter* species associated with *Spartina alterniflora* Loisel roots [2]. It is of phylogenetic interest because of its lifestyle as a symbiotic organism in a marine environment in contrast to many other *Arcobacter* species which are associated with warm-blooded animals and tend to be pathogenic. Here we describe the features of this organism, together with the complete genome sequence, and annotation. This is the first complete genome sequence of a type stain of the genus *Arcobacter.* The 3,192,235 bp genome with its 3,154 protein-coding and 70 RNA genes is part of the *** G****enomic* *** E****ncyclopedia of* *** B****acteria and* *** A****rchaea * project.

## Introduction

Strain CI^T^ (= DSM 7299 = ATCC 33309 = CCUG 15893) is the type strain of the species *Arcobacter nitrofigilis*, which is the type species of the genus *Arcobacter* [1]. Strain CI^T^ was isolated from roots of *Spartina alterniflora* Loisel (cordgrass) growing in salty marshes at the East coast of Canada. It was the first description of an organism in this kind of habitat that belonged to the genus *Campylobacter,* as described based on phenotypic and biochemical traits [2]. The species epithet *nitrofigilis* means 'nitrogen-fixing' and is based on the outstanding characteristic of this species [3]. The new genus *Arcobacter*, meaning 'bow-shaped rod', was introduced in 1991 and its separation from the genus *Campylobacter* was based on DNA-DNA and DNA-rRNA hybridization [1]. Up to now, the genus *Arcobacter* comprises nine species, some of which are associated with warm-blooded animals whereas others are found in marine environments.

Within the *Campylobacteraceae* several whole-genome sequences have already been deciphered: *A. butzleri* strain RM4018 [4] (non type strain) is the only member of the genus *Arcobacter*, as well as genomes from seven species of the genus *Campylobacter*, and *Sulfurospirillum deleyianum* [5].

Only few additional strains belonging to the species *A. nitrofigilis* are known in the literature, with F2176 and F2173 [6] being the closest related ones (99% sequence identity). The type strains of the other species of the genus *Arcobacter* share 93.8-94.6% 16S rRNA sequence identity with strain CI^T^, whereas the type strains from other genera in the family *Campylobacteraceae* share less than 89% sequence identity with strain CI^T^ [7]. There are plenty of phylotypes (uncultured bacteria) known from marine environments such as the ridges flanking crustal fluids in oceanic crust (AY704399, clone FD118-51B-02, 98.6%), sea water from Ishigaki port in Japan (AB262370/-71, 96.4%), a mangrove of the Danshui river estuary of northern Taiwan (DQ234254, 95.8%) [8], costal water in the Bohai Bay, China, (FJ155005, 95.8%), in Black Sea shelf sediments in Romania (AJ271655, 95.8%), or from activated sludge in New Zealand (EU104146, 95.8%). Environmental screens and marine metagenome libraries do not contain more than a handful of sequences with >93% 16S rRNA gene sequence identity indicating a sparse representation of closely related strains in the habitats analyzed (status March 2010). Here we present a summary classification and a set of features for *A. nitrofigilis* strain CI^T^, together with the description of the complete genome sequencing and annotation.

## Classification and features

**Figure 1:**
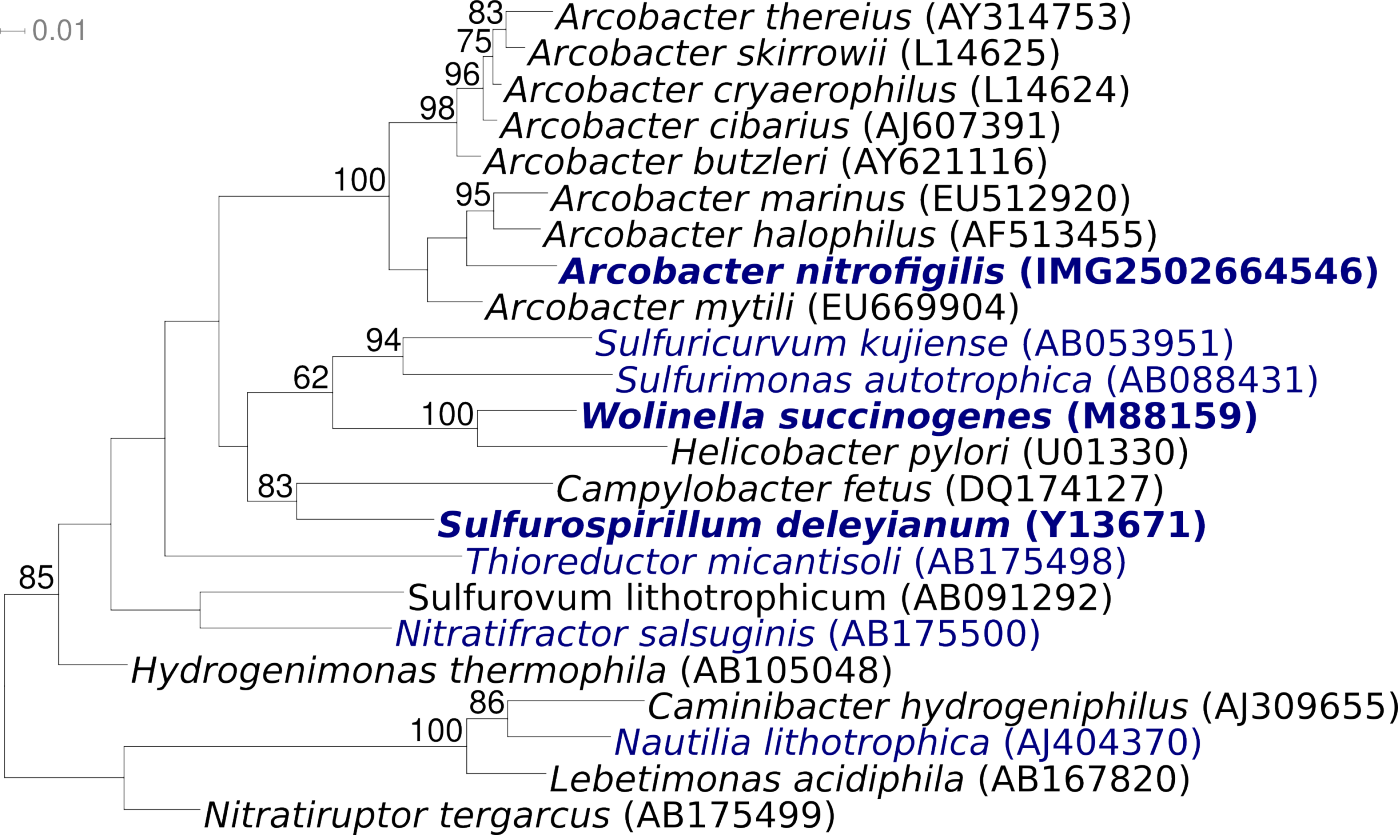
Phylogenetic tree highlighting the position of *A. nitrofigilis* strain CI^T^ relative to the type strains of the other genera within the *Epsilonproteobacteria*. The tree was inferred from 1,379 aligned characters [9,10] of the 16S rRNA gene sequence under the maximum likelihood criterion [11,12] and rooted (as far as possible) in accordance with the current taxonomy [13]. The branches are scaled in terms of the expected number of substitutions per site. Numbers above branches are support values from 200 bootstrap replicates [14] if larger than 60%. Lineages with type strain genome sequencing projects registered in GOLD [15] are shown in blue, published genomes [16] in bold, *e.g*. the recently published GEBA genome from *S. deleyianum* [5].

Figure 1 shows the phylogenetic neighborhood of *A. nitrofigilis* strain CI^T^ in a 16S rRNA based tree. The four 16S rRNA gene sequences in the genome differ from each other by up to two nucleotides, and differ by up to three nucleotides from the previously published 16S rRNA sequence (L14627) generated from CCUG 15893, which contains nine ambiguous base calls.

**Figure 2:**
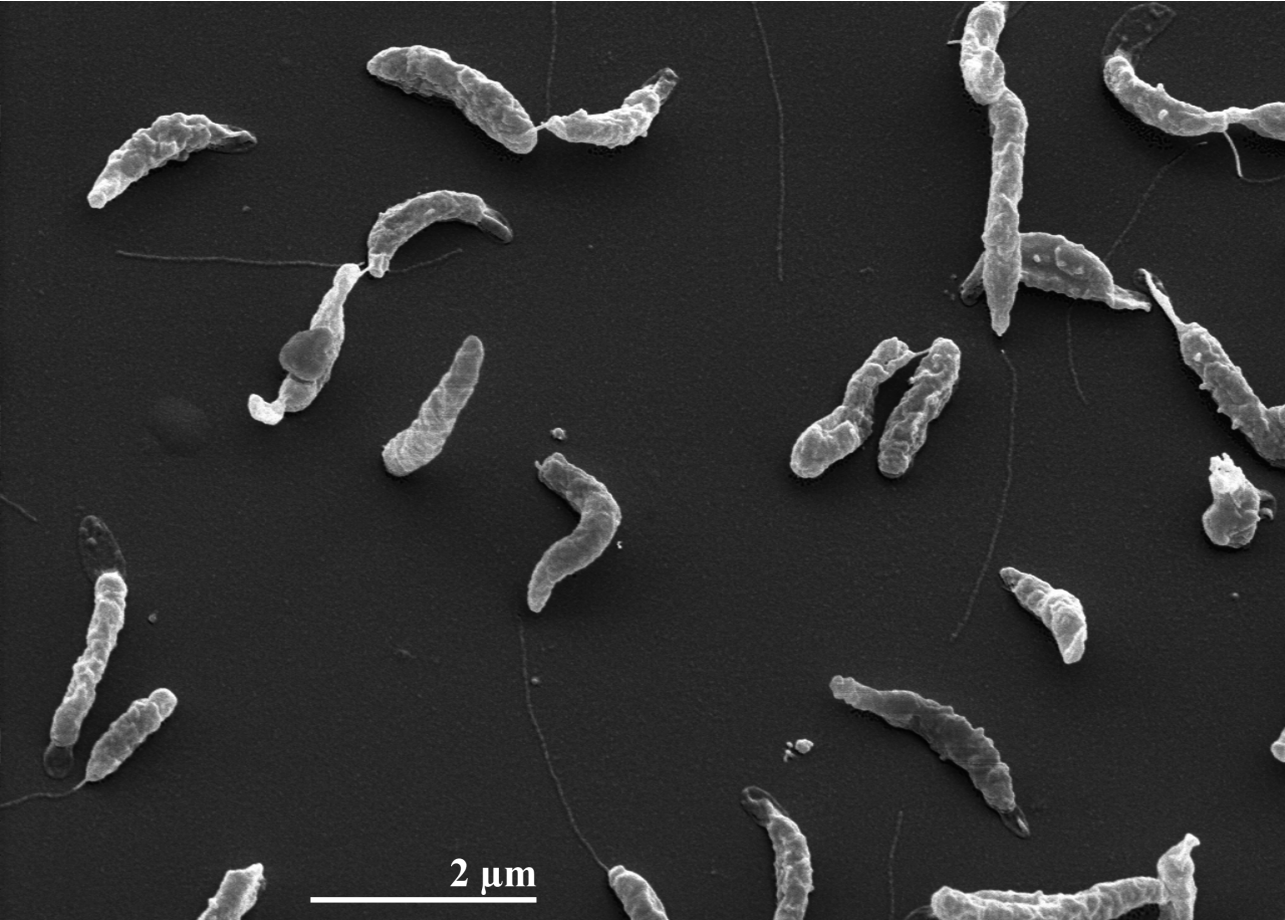
Scanning electron micrograph of *A. nitrofigilis* strain CI^T^

*A. nitrofigilis* cells are Gram-negative, bow-shaped or curved rods of 1–3 µm length and 0.2–0.9 µm width (Figure 2 and Table 1). Motility is based on a single, polar flagellum and results in rapid corkscrew motion. Older cultures also show coccoid cells [2]. The habitat of all known *A. nitrofigilis* isolates is either the roots or the sediment around the roots of *S. alterniflora* Loisel growing in salt marshes [3]. Although no pathogenic association has been described so far, *A. nitrofigilis* was among five *Arcobacter* species that were isolated from food samples such as meat and shellfish varieties [27]. The optimum growth temperature of *A. nitrofigilis* is 30°C, the temperature range is from 10–37°C [28]. Neither spores nor granules are present but a brown pigment is formed from tryptophan [2]. All strains of the species show positive reactions for nitrogenase, catalase and oxidase. Growth occurs under microaerophilic conditions with oxygen as terminal electron acceptor, under anaerobic conditions fumarate or aspartate are necessary, the presence of nitrate is detrimental [2]. Hydrogen is not necessary for growth [1]. Nitrate is reduced to nitrite and sulfide is produced from cysteine [3]. Strain CI^T^ tested positive for urease, other strains of the species do not [3]. The metabolism of *A. nitrofigilis* is chemoorganotrophic; organic acids and amino acids are used as carbon sources but carbohydrates are neither oxidized nor fermented [2]. All strains of the species are halotolerant. They require a minimum of 0.5% NaCl for growth and can tolerate up to 7% NaCl [28]. *A. nitrofigilis* is susceptible to cephalothin and nalidixic acid but isresistant to vancomycin [3]. The G+C content of the DNA was determined by thermal denaturation to be 28.0% [3] which is slightly below the 28.4% found in the genome.

**Table 1:** Classification and general features of *A. nitrofigilis* strain CI^T^ according to the MIGS recommendations [17]

**MIGS ID**	**Property**	**Term**	**Evidence code**
	Current classification	Domain *Bacteria*	TAS [18]
		Phylum ‘*Proteobacteria*’	TAS [19]
		Class *Epsilonproteobacteria*	TAS [20,21]
		Order *Campylobacterales*	TAS [20,22]
		Family *Campylobacteraceae*	TAS [23]
		Genus *Arcobacter*	TAS [1]
		Species *Arcobacter nitrofigilis*	TAS [1]
		Type strain CI	TAS [3]
	Gram stain	negative	TAS [2]
	Cell shape	bow-shaped rods	TAS [2]
	Motility	motile	TAS [2]
	Sporulation	non-sporulating	TAS [2]
	Temperature range	mesophile, 10-37°C	TAS [2]
	Optimum temperature	30°C	TAS [24]
	Salinity	halotolerant up to 7% NaCl	TAS [2]
MIGS-22	Oxygen requirement	microaerophilic	TAS [2]
	Carbon source	organic and amino acids	TAS [1]
	Energy source	chemoorganotroph	TAS [3]
MIGS-6	Habitat	marine	TAS [2]
MIGS-15	Biotic relationship	symbiotic	TAS [2]
MIGS-14	Pathogenicity	none	NAS
	Biosafety level	1	TAS [25]
	Isolation	roots of the marshplant *Spartina alterniflora*	TAS [2]
MIGS-4	Geographic location	Conrads Beach (Dartmouth), Nova Scotia (Canada)	TAS [2]
MIGS-5	Sample collection time	about or before 1980	TAS [2]
MIGS-4.1 MIGS-4.2	Latitude Longitude	44.65 -63.60	NAS
MIGS-4.3	Depth	unknown	
MIGS-4.4	Altitude	sea level	

Evidence codes - IDA: Inferred from Direct Assay (first time in publication); TAS: Traceable Author Statement (i.e., a direct report exists in the literature); NAS: Non-traceable Author Statement (i.e., not directly observed for the living, isolated sample, but based on a generally accepted property for the species, or anecdotal evidence). These evidence codes are from of the Gene Ontology project [26]. If the evidence code is IDA, then the property was directly observed by one of the authors or an expert mentioned in the acknowledgements.

### Chemotaxonomy

The major respiratory quinones are menaquinone 6 and a second atypical menaquinone 6 that has not yet been clearly identified [1]. The major fatty acids in whole cells of *A. nitrofigilis* are hexadecenoic (C_16:0_), cis-9-hexadecenoic (cis-C_16:1ϖ7c_) and cis-9-octadecenoic acid (cis-C_18:1ϖ7c_) [24]

## Genome sequencing and annotation information

### Genome project history

This organism was selected for sequencing on the basis of its phylogenetic position [29], and is part of the *** G****enomic* *** E****ncyclopedia of* *** B****acteria and* *** A****rchaea * project [30]. The genome project is deposited in the Genomes OnLine Database [15] and the complete genome sequence in GenBank. Sequencing, finishing and annotation were performed by the DOE Joint Genome Institute (JGI). A summary of the project information is shown in Table 2.

**Table 2:** Genome sequencing project information

**MIGS ID**	**Property**	**Term**
MIGS-31	Finishing quality	Finished
MIGS-28	Libraries used	Three genomic libraries: 454 pyro-sequence standard library, 454 pyro-sequence 24 kb PE library, and Illumina stdandard library
MIGS-29	Sequencing platforms	454 GS FLX, Illumina GAii
MIGS-31.2	Sequencing coverage	43.5× pyrosequence, 15.7× Illumina
MIGS-30	Assemblers	Newbler version 2.0.0- PostRelease-10/28/2008, phrap
MIGS-32	Gene calling method	Prodigal 1.4, GenePRIMP
	INSDC ID	CP001999
	Genbank Date of Release	May 18, 2010
	GOLD ID	Gc01280
	NCBI project ID	32593
	Database: IMG-GEBA	2502545034
MIGS-13	Source material identifier	DSM 7299
	Project relevance	Tree of Life, GEBA

### Growth conditions and DNA isolation

*A. nitrofigilis* strain CI^T^, DSM 7299, was grown on DSMZ medium 429 (Columbia agar including 5% horse blood) [31] at 28°C. DNA was isolated from 1-1.5 g of cell paste using Qiagen Genomic 500 DNA Kit (Qiagen, Hilden, Germany) with lysis modification st/LALMP according to Wu *et al*. [30].

### Genome sequencing and assembly

The genome was sequenced using a combination of Illumina and 454 technologies. An Illumina GAii shotgun library with reads of 50 Mb, a 454 Titanium draft library with average read length of 243 bases, and a paired end 454 library with average insert size of 24 kb were generated for this genome. All general aspects of library construction and sequencing can be found at http://www.jgi.doe.gov/. Draft assembly was based on 138 Mb 454 standard and 454 paired end data (498,215 reads). Newbler (Roch, version 2.0.0-PostRelease-10/28/2008) parameters are -consed -a 50 -l 350 -g -m -ml 20. The initial Newbler assembly contained 42 contigs in 3 scaffolds. It was converted into a phrap assembly by making fake reads from the consensus and collecting the read pairs in the 454 paired end library. Illumina sequencing data was assembled with Velvet [32], and the consensus sequences were shredded into 1.5 kb overlapped fake reads and assembled together with the 454 data. The Phred/Phrap/Consed software package (www.phrap.com) was used for sequence assembly and quality assessment in the following finishing process. After the shotgun stage, reads were assembled with parallel phrap (High Performance Software, LLC). Possible mis-assemblies were corrected with gapResolution (http://www.jgi.doe.gov/), Dupfinisher, or sequencing cloned bridging PCR fragments with subcloning or transposon bombing [33]. Gaps between contigs were closed by editing in Consed, by PCR and by Bubble PCR primer walks (J-F.Cheng, unpublished). A total of 480 additional Sanger reactions were necessary to close gaps and to raise the quality of the finished sequence. Illumina reads were also used to improve the final consensus quality using an in-house developed tool (the Polisher). The error rate of the completed genome sequence is less than 1 in 100,000.

### Genome annotation

Genes were identified using Prodigal [34] as part of the Oak Ridge National Laboratory genome annotation pipeline, followed by a round of manual curation using the JGI GenePRIMP pipeline [35]. The predicted CDSs were translated and used to search the National Center for Biotechnology Information (NCBI) nonredundant database, UniProt, TIGR-Fam, Pfam, PRIAM, KEGG, COG, and InterPro databases. Additional gene prediction analysis and functional annotation was performed within the Integrated Microbial Genomes - Expert Review (IMG-ER) platform [36].

## Genome properties

**Figure 3:**
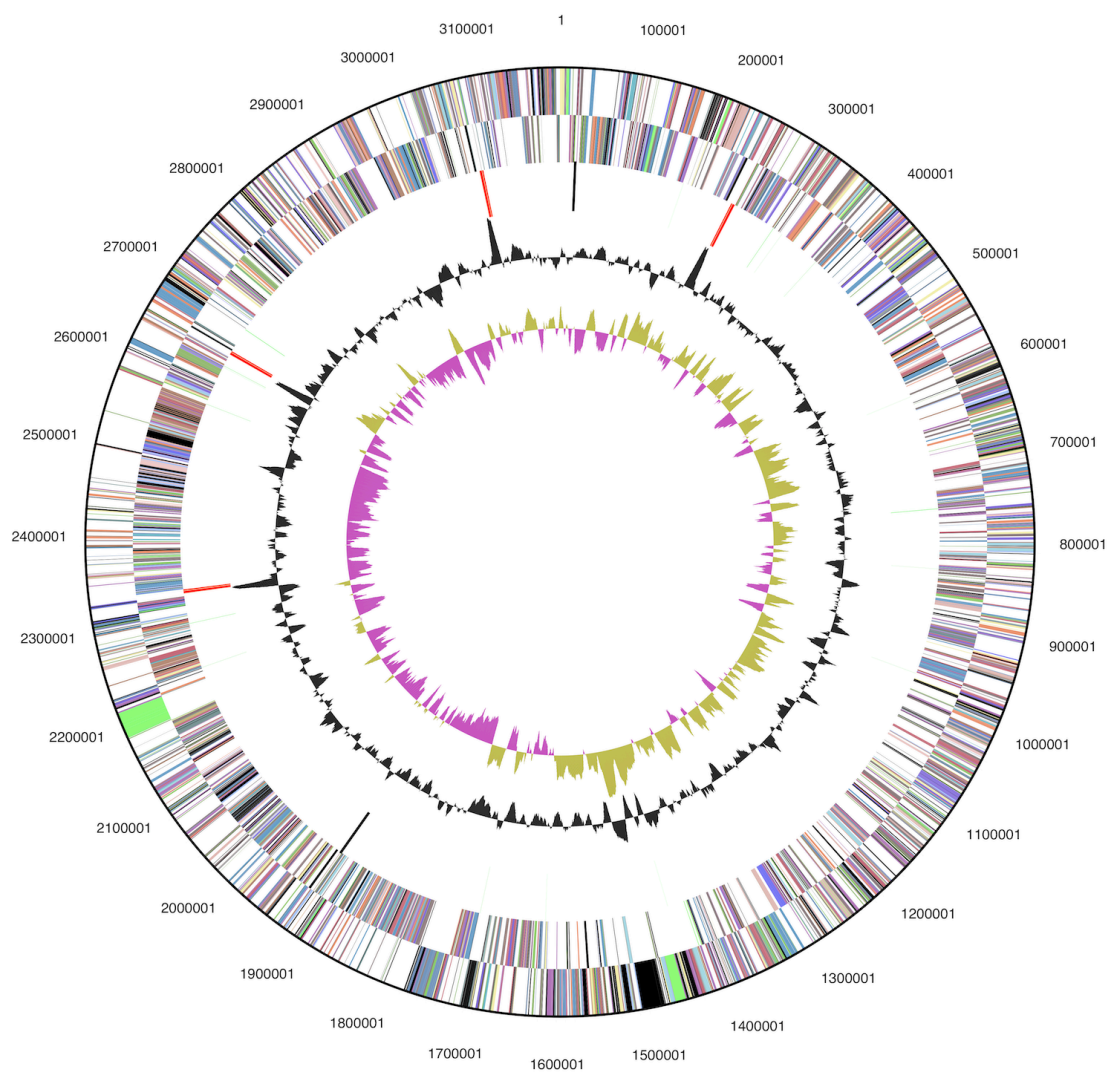
Graphical circular map of the chromosome. From outside to the center: Genes on forward strand (color by COG categories), Genes on reverse strand (color by COG categories), RNA genes (tRNAs green, rRNAs red, other RNAs black), GC content, GC skew.

The genome is 3,192,235 bp long and comprises one main circular chromosome with an overall G+C content of 28.4% (Table 3 and Figure 3). Of the 3,224 genes predicted, 3,154 were protein-coding genes, and 70 RNAs; 28 pseudogenes were also identified. The majority of the protein-coding genes (72.1%) were assigned a putative function while those remaining were annotated as hypothetical proteins. The distribution of genes into COGs functional categories is presented in Table 4.

**Table 3:** Genome Statistics

**Attribute**	**Value**	**% of Total**
Genome size (bp)	3,192,235	100.00%
DNA coding region (bp)	3,009,967	94.29%
DNA G+C content (bp)	905,345	28.36%
Number of replicons	1	
Extrachromosomal elements	0	
Total genes	3,224	100.00%
RNA genes	70	2.17%
rRNA operons	4	
Protein-coding genes	3,154	97.83%
Pseudo genes	70	2.17%
Genes with function prediction	2,324	72.08%
Genes in paralog clusters	454	14.08%
Genes assigned to COGs	2,363	73.29%
Genes assigned Pfam domains	2,480	76.92%
Genes with signal peptides	597	18.52%
Genes with transmembrane helices	838	25.99%
CRISPR repeats	1	

**Table 4:** Number of genes associated with the general COG functional categories

**Code**	**value**	**%age**	**Description**
J	143	5.4	Translation, ribosomal structure and biogenesis
A	0	0.0	RNA processing and modification
K	157	5.9	Transcription
L	102	3.9	Replication, recombination and repair
B	0	0.0	Chromatin structure and dynamics
D	16	0.6	Cell cycle control, mitosis and meiosis
Y	0	0.0	Nuclear structure
V	37	1.4	Defense mechanisms
T	267	10.1	Signal transduction mechanisms
M	168	6.3	Cell wall/membrane/envelope biogenesis
N	78	3.0	Cell motility
Z	0	0.0	Cytoskeleton
W	0	0.0	Extracellular structures
U	69	2.6	Intracellular trafficking and secretion
O	103	3.9	Posttranslational modification, protein turnover, chaperones
C	212	8.0	Energy production and conversion
G	114	4.3	Carbohydrate transport and metabolism
E	252	9.5	Amino acid transport and metabolism
F	61	2.3	Nucleotide transport and metabolism
H	128	4.8	Coenzyme transport and metabolism
I	57	2.2	Lipid transport and metabolism
P	159	6.0	Inorganic ion transport and metabolism
Q	38	1.4	Secondary metabolites biosynthesis, transport and catabolism
R	288	10.9	General function prediction only
S	199	7.5	Function unknown
-	861	26.1	Not in COGs
